# Conformational preferences of fluorine-containing agrochemicals and their implications for lipophilicity prediction

**DOI:** 10.3762/bjoc.16.200

**Published:** 2020-10-05

**Authors:** Daniela Rodrigues Silva, Joyce K Daré, Matheus P Freitas

**Affiliations:** 1 Department of Chemistry, Federal University of Lavras, 37200–900, Lavras–MG, Brazil

**Keywords:** dipole moment, fluorinated compounds, *gauche* effect, herbicides, log *P*

## Abstract

Molecular polarity governs lipophilicity, which in turn determines important agrochemical and environmental properties, such as soil sorption and bioconcentration of organic compounds. Since the C–F bond is the most polar in organic chemistry, the orientation of fluorine substituents originating from the rotation around C–C(F) bonds should affect the polarity and, consequently, the physicochemical and biological properties of fluorine-containing agrochemicals. Accordingly, this study aims to determine the most likely conformers of some fluorine-containing agrochemicals and to correlate their molecular dipole moments with the respective *n*-octanol/water partition coefficients (log *P*), in order to investigate the dependence of the lipophilicity with the molecular conformation.

## Introduction

Whilst in the last years the agrochemical industry has encountered a period of downturn affected by new regulations, low crop prices, biochemical resistance, among other variables, recent events have shown signs of recovery [1]. Currently, the agrochemical industry focuses on introducing new efficient and more environmentally friendly products, that attend the new regulation requirements, for replacing those agrochemicals that were banned due to either their hazard or inefficiency in fighting persistent weeds and pathogens [1]. However, the process for designing, developing, and introducing new agrochemicals to the market is considerably challenging, since it involves many steps and the optimization of a range of properties. Furthermore, it is also an expensive and time-consuming procedure [2].

Fluorination is a common strategy employed as part of the optimization process during the design of new chemical compounds, which includes the modulation of a variety of properties such as lipophilicity, biological half-life, and biosorption [3–4]. This role helps in explaining the expressive amount of fluorine-containing agrochemical candidates (around 30%) as well as pharmaceuticals (around 20%) [5–6]. In this sense, the chemistry of fluorine-containing compounds has been extensively investigated in order to better understand the effects of fluorination on conformation, membrane permeation, pharmacokinetic properties, among other parameters [7].

From a conformational analysis point of view, the fluorine atom presents minimal steric effects; on the other hand, due to its high electronegativity, the C–F bond is highly polarized, which characterizes it as a site for electrostatic and hyperconjugative interactions [8]. Additionally, Juaristi and Notario [9] and O’Hagan and co-workers [10] have highlighted the crucial role of hyperconjugative interactions involving the fluorine lone pair in explaining the chemical behavior of organofluorine molecules and their unusual physicochemical properties. Lastly, Müller [11] explained that these features along with fluorine’s strict monovalent binding mode and little polarizability, when covalently bound, guarantee fluorination the well-known ability of modulating physicochemical properties.

Although the stereochemical effects of fluorination, responsible for specific interactions and conformational preferences of several groups of compounds, have become increasingly well understood [12], their direct implication on physicochemical properties has not been fully investigated yet. The orientation of fluorine substituents originated from the rotation of fluorine-containing C–C bonds should affect the polarity and, therefore, the physicochemical and biological properties of organofluorine compounds. However, there is a lack of studies that explain how these well-known conformational effects directly alter macroscopic observed properties, such as lipophilicity [13].

**Figure 1:**
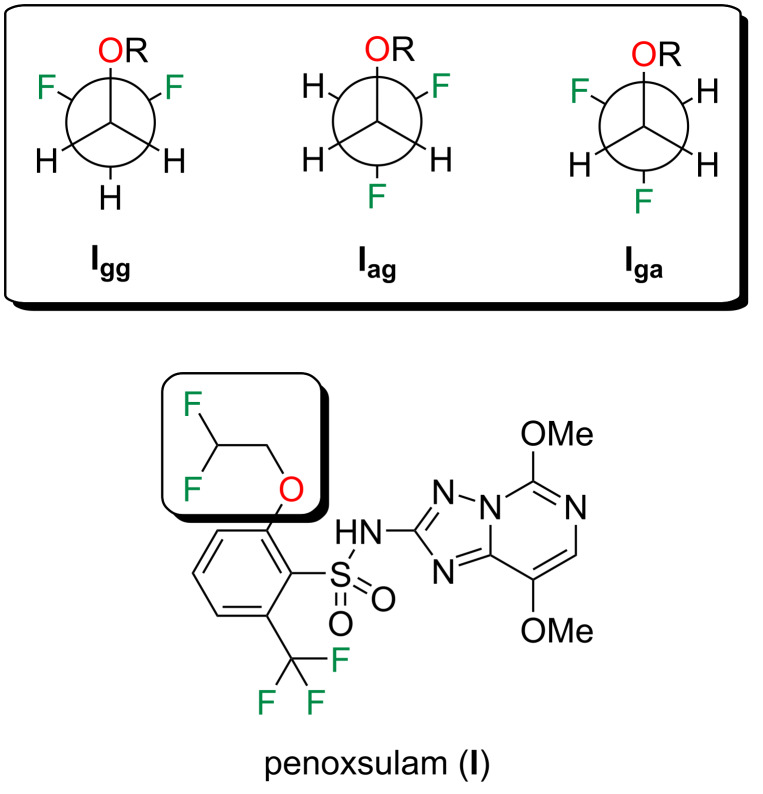
Chemical structure of penoxsulam (**I**) and the main staggered conformations along the two F–C–C–O torsional angles (i.e., **I_gg_**, **I_ag_**, and **I_ga_**; g = *gauche* and a = *anti*).

Accordingly, the main goal of this work is to investigate the relationship between lipophilicity and molecular conformation on a set of organofluorine agrochemicals. To this end, this study has been divided in two parts. First, we have analyzed the conformational equilibrium of penoxsulam (**I**, Figure 1). This compound has a 1,2–disubstituted ethane motif that could adopt three main staggered conformations, namely **I_gg_**, **I_ag_** and **I_ga_** (g = *gauche* and a = *anti*; see Figure 1), thus we have explored the intramolecular interactions governing its conformational preferences. It is worth mentioning that **I_gg_** has two *gauche* relationships between C–F and C–O bonds, which is stabilized by σ_CH_ → σ_CF/CO_ hyperconjugative interactions [14]. Second, we have searched for the implications of the relative conformational stabilities for log *P* prediction, the most common parameter employed to describe lipophilicity. It has been previously established that the lipophilicity of a compound depends greatly on the overall molecular polarity [15], which is often expressed as the molecular dipole moment (μ). In turn, the orientation of polar bonds also influences the overall polarity of an organic molecule, as above mentioned. Therefore, this work also seeks to assess a correlation between calculated μ values and experimental log *P* measures, in order to unveil the dependence of lipophilicity with molecular conformation.

## Results and Discussion

### Conformational analysis of penoxsulam

**Figure 2:**
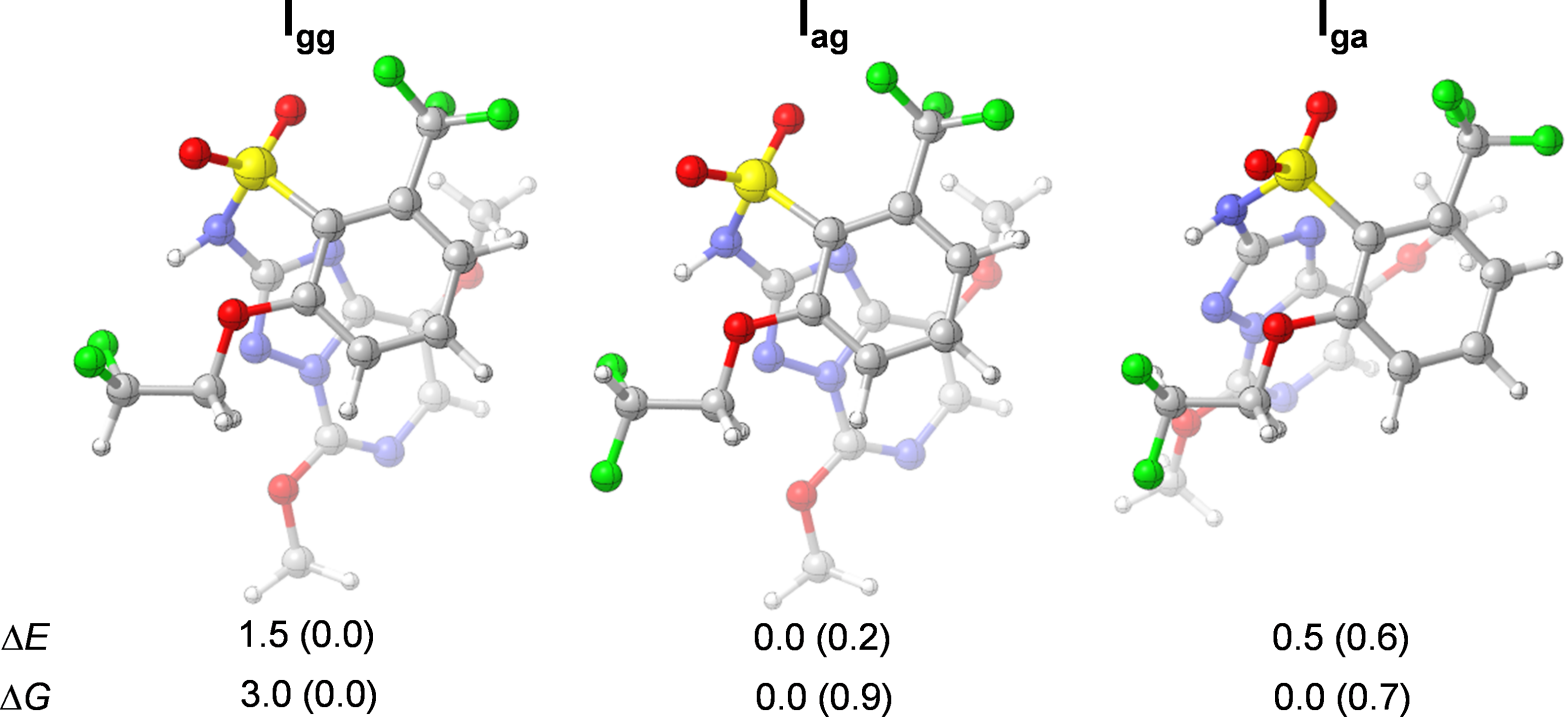
Optimized structures of conformers **I_gg_** (left), **I_ag_** (middle), and **I_ga_** (right), along with the relative electronic and Gibbs free energies (in kcal mol^−1^) in the gas phase and in water solution (in parentheses), computed at the ωB97X-D/6-311++G(d,p) level of theory.

Given the high degree of freedom in the chemical structure of penoxsulam (**I**), the conformational analysis started with a Monte Carlo conformational search at the ωB97X-D/6-31G(d,p) [16–17] level of the density functional theory (DFT). The global energy minimum conformation was then re-optimized in a higher level of theory, ωB97X-D/6-311++G(d,p) [16,18], resulting in conformer **I_ag_** (Figure 2). This conformer has one fluorine atom in an *anti*-orientation and the other one in a *gauche*-orientation relative to the vicinal oxygen atom (pointing towards the amine hydrogen atom). To evaluate the other possible conformations along the 1,2-disubstituted ethane motif, the C–C(F) bond was rotated to additionally obtain conformers **I_gg_** and **I_ga_**, and the corresponding geometries were then optimized. The resulting geometries and relative conformational energies are summarized in Figure 2.

At first, we observe that the overall geometry is quite similar among the three conformers. Only for **I_ga_**, where the *gauche* fluorine atom points to the opposite direction of the amine group, the triazole ring was farther away from the 1,2-disubstituted ethane moiety. Note that the gas-phase relative conformational energy Δ*E* increases, i.e., becomes less stabilizing, in the order **I_ag_** < **I_ga_** < **I_gg_**. The difference in energy between **I_ag_** and **I_ga_** is somehow small (0.5 kcal mol^−1^), and these conformers are equally stable according to the relative Gibbs free energy Δ*G* (Boltzmann populations of 50% and 49%, respectively). The inclusion of an implicit polar solvent (e.g. water), however, decreases the difference in energy among conformers and **I_gg_** becomes the most stable conformer in solution, i.e., a double *gauche* effect takes place (see data in parentheses in Figure 2). This is not surprising, since **I_gg_** has the highest dipole moment (data shown in the next section) and is naturally more stabilized by polar solvents. Therefore, further analysis will consider the gas phase, since in this way we are accounting for the intrinsic intramolecular interactions without the influence of solvent as an external factor.

**Figure 3:**
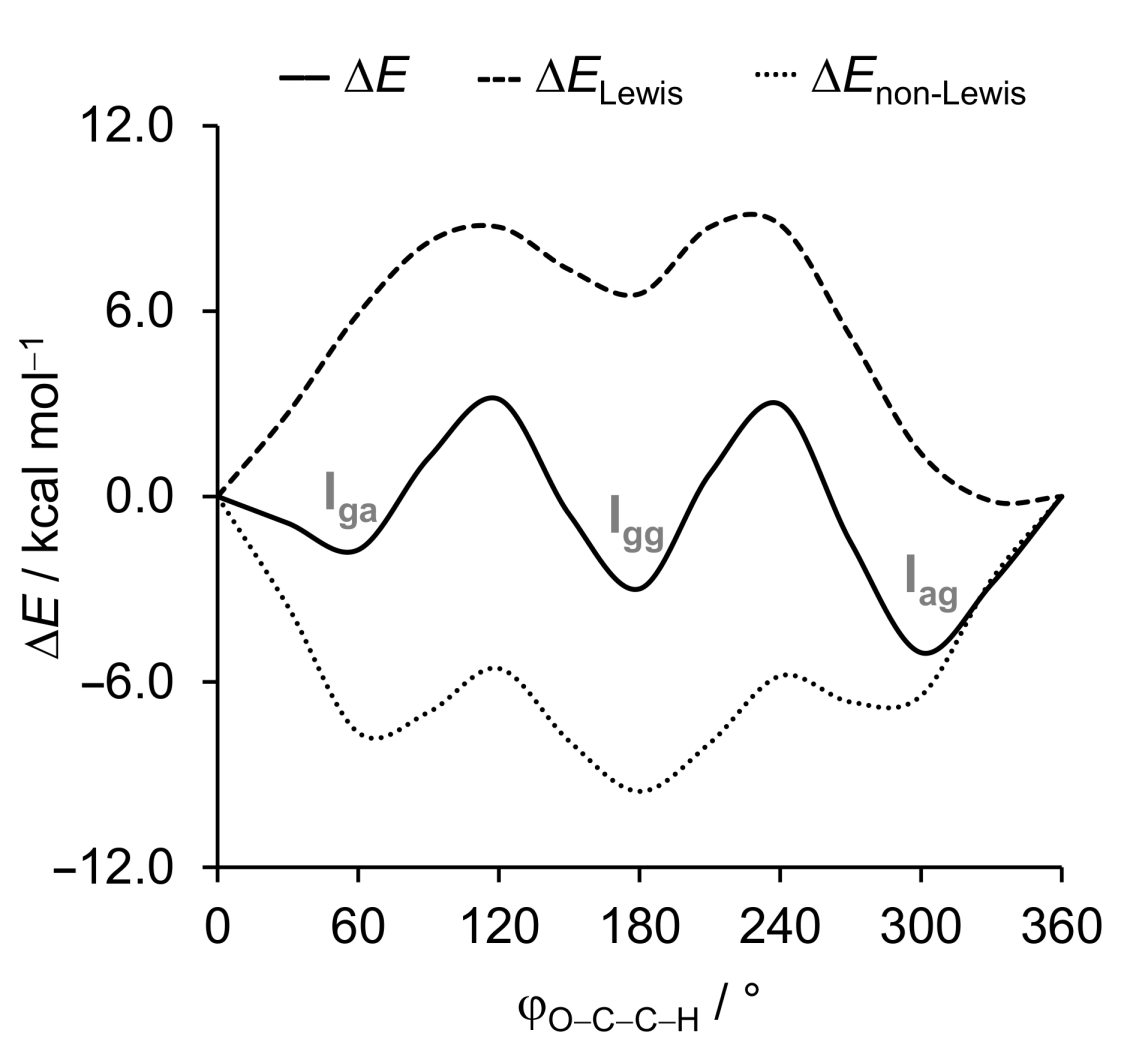
Energy profile for the rotation around the C–C(F) bond and NBO analysis project onto the φ_O–C–C–H_ torsional angle (step size of 30°) at the ωB97X-D/6-311G(d,p) level of theory.

To better understand the relative conformational stabilities, we performed a numerical experiment in which the C–C(F) bond is rotated from conformer **I_ag_** keeping other geometrical parameters fixed. In this way, we can specifically investigate the intramolecular interactions governing conformational preferences in the 1,2–disubstituted ethane motif. Furthermore, we decomposed the conformational energy along rotation around the C–C(F) bond within the framework of the natural bond orbital (NBO) analysis [19] into the Lewis (Δ*E*_L_, which accounts for classical interactions) and non-Lewis (Δ*E*_NL_, which accounts for delocalization energy) contributions (Figure 3). Note that all energy terms are represented relative to the conformation with the φ_O–C–C–H_ torsional angle of 0°, thus positive values mean that the energy becomes less stabilizing and negative values mean that the energy becomes more stabilizing.

From Figure 3, it can be observed that conformer **I_gg_** is the most stabilized by the Δ*E*_NL_ term, which can be attributed to the stabilizing interactions featured in the *gauche* effect [14,20], due to the *gauche* arrangement along the two F–C–C–O pathways. The charge transfer from the filled σ_CH_ orbital to the empty σ*_CF_ or σ*_CO_ orbitals amounts to 4.6/4.5 and 3.3 kcal mol^−1^, respectively, which are more stabilizing than the corresponding σ_CF/CO_ → σ*_CO/CF_ and n_F/O_ → σ*_CO/CF_ in the *anti*-orientation (Table 1). Note that the n_F_ → σ*_CO_ charge transfer is slightly more stabilizing than the σ_CF_ → σ*_CO_, which is in agreement with the findings of Juaristi and Notario [9], and O’Hagan and co-workers [10]. Besides, there is also an interaction between the fluorine electron lone pair n_F_ and the σ*_NH_ orbital of the amine group (of 3.2 kcal mol^−1^, see Table 1). **I_ga_** also experiences this stabilizing hydrogen bond-like intramolecular interaction (n_F_ → σ_NH_ of 2.8 kcal mol^–1^, see Table 1). Yet, conformer **I_gg_** is not the global energy minimum. This is surprising, because all-*gauche* conformations in fluoropropanediol are strongly preferred [21], while the double *gauche* effect in difluoroethylamine and its hydrochloride salt stabilizes the gg over ag conformations [22]. Therefore, **I_ag_** is the global energy minimum because it experiences a more stabilizing Δ*E*_L_ term. Among the three conformers, **I_gg_** has the least stabilizing Δ*E*_L_ energy, and this can be ascribed to the closer proximity between the F and O electronegative atoms. Thus, the least stabilizing Δ*E*_L_ term overcomes the stabilization from hyperconjugation interactions (Δ*E*_NL_), and classical electrostatic and steric interactions are the main factors governing conformational preferences of penoxsulam (**I**).

**Table 1:** Second order perturbation energy *E*(2) of the main hyperconjugation interactions (in kcal mol^–1^) computed at the ωB97X-D/6-311G(d,p) level of theory.

Conf.	σ_CH_ → σ*_CF_	σ_CH_ → σ*_CO_	σ_CO_ → σ*_CF_	σ_CF_ → σ*_CO_	n_F_ → σ*_CO_	n_O_ → σ*_CF_	n_F_ → σ*_NH_

**I_gg_**	4.6/4.5	3.3	–	–	–	–	3.2
**I_ag_**	4.7	–	1.5	1.5	1.8	0.8	2.8
**I_ga_**	4.3	–	1.7	1.4	1.6	0.8	–

It is worth mentioning that the structure of penoxsulam in the biological environment is already known in the literature [23] and, accordingly, it differs from the energy minimum conformations computed in this work. However, a conformational search in the gas phase, as performed herein, is necessary to fully understand the intramolecular interactions and to establish the correlation between μ and log *P*, since this physicochemical property does not depend on the geometry of penoxsulam inside a biological receptor.

### Effect of molecular conformation on log *P*

Herein, we aim at evaluating the correlation of molecular conformation with lipophilicity, described in terms of the *n*-octanol/water partition coefficient – log *P*, through molecular dipole moment (μ). The molecular dipole moment, μ is a relatively simple parameter that can inform on subtle intramolecular interactions that favor one structural arrangement over another, as previously mentioned. Therefore, by using a weighted average μ over the most likely conformations to correlate with experimental log *P*, one can assess the dependence of log *P* with molecular conformation.

**Figure 4:**
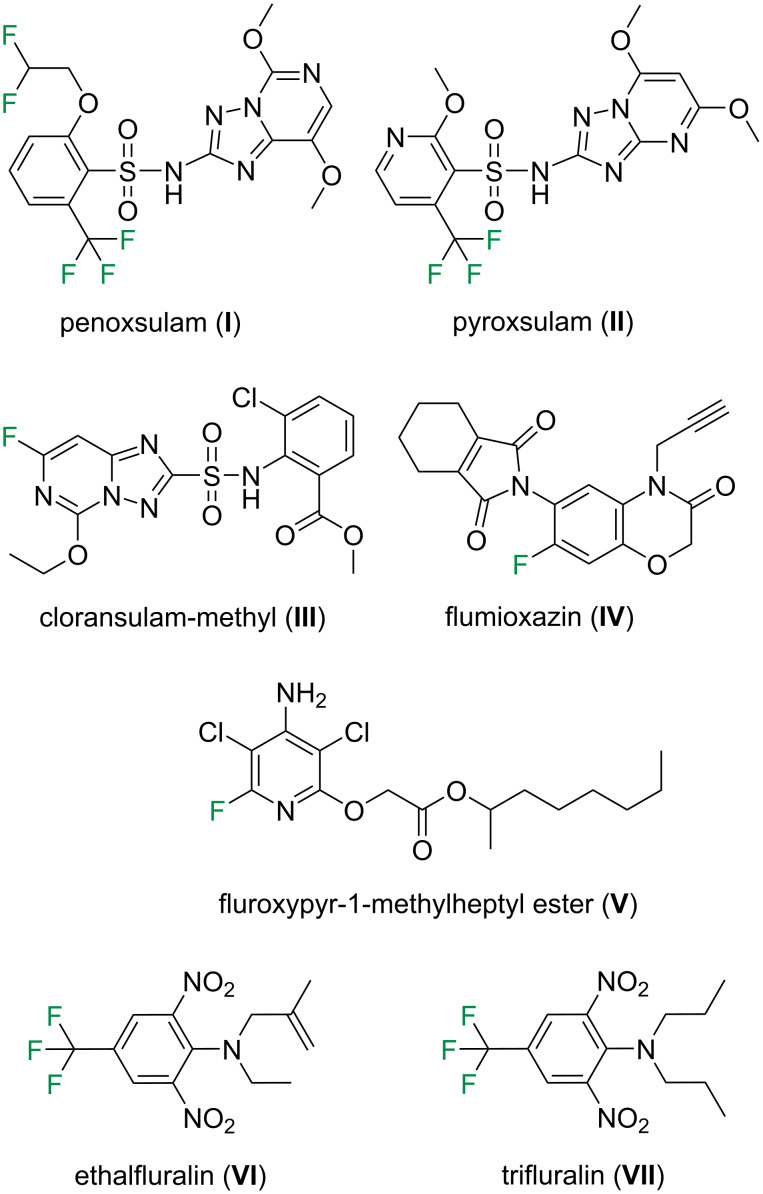
Chemical structure of the agrochemicals **I**–**VII** analyzed herein.

Accordingly, a consistent set of fluorine-containing agrochemicals (Figure 4) with experimentally available log *P* data was selected from a single database [24], which comprises: penoxsulam (**I**), pyroxsulam (**II**), cloransulam-methyl (**III**), flumioxazin (**IV**), fluroxypyr-1-methylheptyl ester (**V**), ethalfluralin (**VI**), and trifluralin (**VII**). The data set contains compounds without rotatable C–C(F) bonds (**III**–**V**), with a rotatable C–C(F) bond that does not generate different conformers (**II**, **VI**, and **VII**), and with a rotatable C–C(F) bond that generates different conformers (**I**). The μ values for all herbicides were computed through theoretical calculations (see computational details section) and are presented in Table 2 along with their respective experimental log *P* data.

**Table 2:** Experimental log *P*, dipole moment (μ, in Db), and predicted log *P* of agrochemicals **I**–**VII**.

Compd.	log *P*^a^	μ^b^	milog *P*^c^

**I_gg _[0.01]**	−0.60	11.56	2.74
**I_ag _[0.50]**	−0.60	9.61	2.74
**I_ga _[0.49]**	−0.60	9.50	2.74
**II**	−1.01	7.24	1.44
**III**	1.12	7.76	3.09
**IV**	2.55	4.27	2.12
**V**	5.04	0.68	5.43
**VI**	5.11	2.32	4.28
**VII**	5.27	2.85	4.47

^a^Experimental log *P* obtained from the Dow AgroSciences database [24]; ^b^dipole moments computed at the ωB97X-D/6-311++G(d,p) level of theory, see computational section for details; ^c^predicted log *P* calculated in the Molinspiration Cheminformatics server [25].

**Figure 5:**
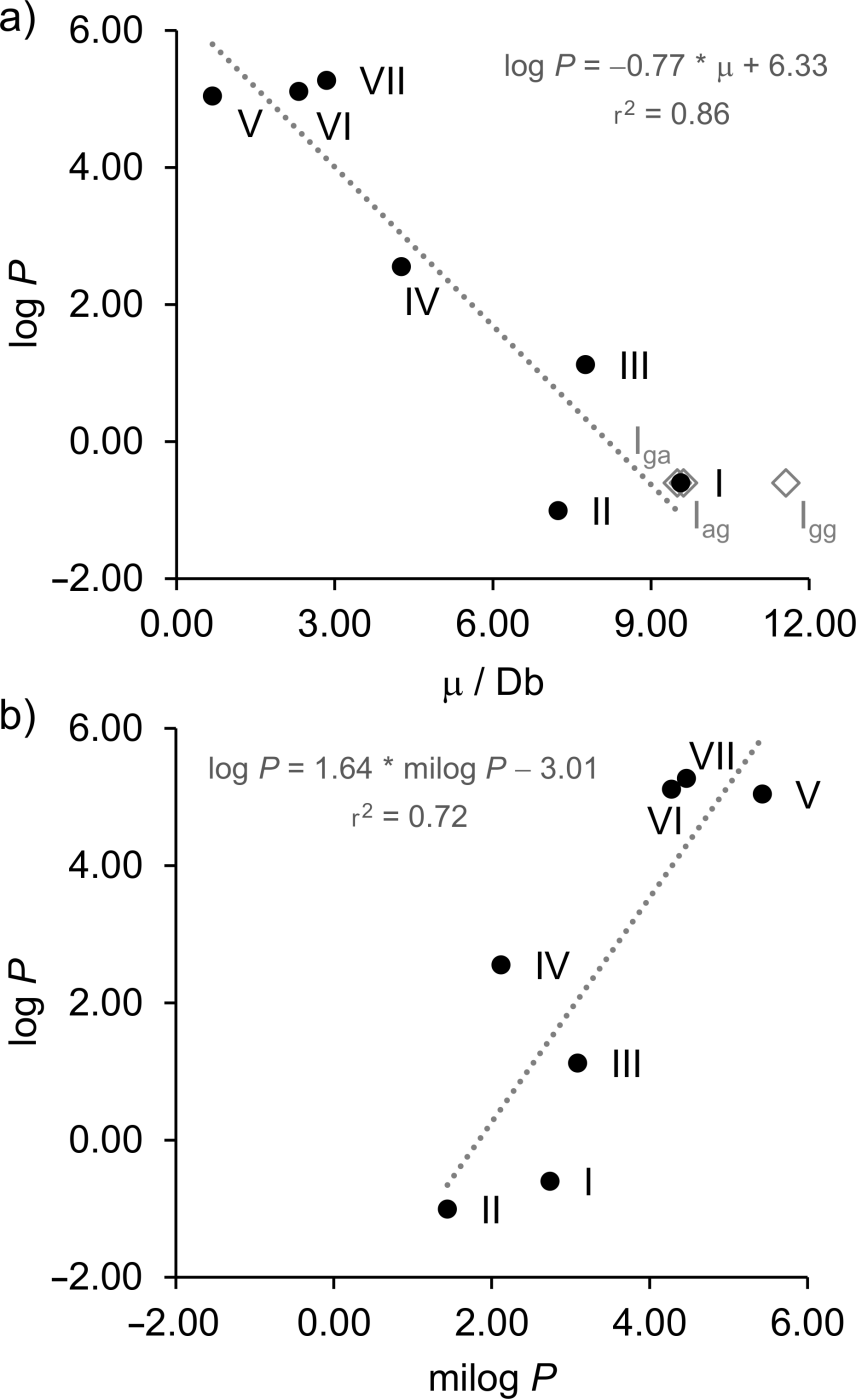
Correlation between the experimental log *P* of agrochemicals **I**–**VII** and a) dipole moment (for **I**, μ is used as a weighted average of the conformers **I_gg_**, **I_ag_**, and **I_ga_**), and b) predicted log *P* (milog *P*).

Penoxsulam (**I**) has a rotatable C–C(F) bond (as analyzed in the previous section) and, consequently, has the dependence of the molecular dipole moment μ with the rotation around this bond. It is well-known that μ is influenced by two main factors: the molecular dimensions and the electron distribution [26]. Thus, a different μ was calculated for each of the three staggered conformers of **I** and a weighted average μ was obtained from the calculated μ of the three conformers **I_gg_**, **I_ag_**, and **I_ga_**. Then, the calculated μ values were plotted against the experimental log *P* (Figure 5a) in order to quantitatively analyze the correlation between the two parameters. The resulting linear regression coefficient (r^2^) was 0.86, which can be considered a suitable correlation coefficient. A hypothesis test for the significance of the observed correlation, based on the *p*-value (at a significance level of 95%, α = 0.05), was also carried out. A *p*-value of 0.00264 was obtained, which is much smaller than the critical value of 0.05. Therefore, the null hypothesis (that considers the correlation coefficient as 0) is rejected, which reinforces the dependence of lipophilicity on the total molecular polarity.

In another attempt to demonstrate the importance of μ (a conformational dependent parameter) for estimating log *P*, we employed the Molinspiration Cheminformatics tool [25] to predict log *P*, and the results are displayed in Table 2. The predicted outcomes were also plotted against the experimental values (see Figure 5b). Most tools for predicting physicochemical properties are based on the additive contributions of polarity from atoms or chemical groups instead of considering the overall molecular polarity (μ) [27]. The predictive ability of these methods for compounds with higher structural complexity have been questioned in the literature [28]. Indeed, the obtained r^2^ (0.72) exhibits a considerably higher discrepancy than that correlation obtained from μ when compared to the optimal value of 1. A linear regression of experimental log *P* against calculated log *P* values obtained from another source (the ChemSketch module of the ACD/Labs program) yields a similar result (r^2^ = 0.71, see Figure S1 in Supporting Information File 1). Moreover, from Figure 5b, due to the high slope and the resulting intercept, one can assume the existence of a systematic error associated with the prediction of log *P*. This finding reinsures the issues with additive methods for predicting lipophilicity of complex structures as those analyzed herein.

Nevertheless, a reservation should be considered for small organofluorine compounds, for which well-parameterized models for log *P* prediction are usually available.

**Figure 6:**
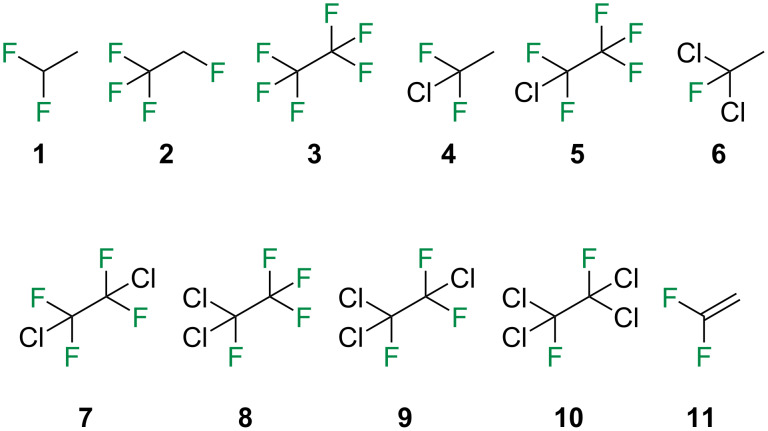
Chemical structure of the compounds **1**–**11** analyzed herein.

Regarding the use of calculated molecular dipole moments as a descriptor of lipophilicity for small molecules, a more detailed analysis is required. Accordingly, a series of structurally simpler organofluorine compounds were retrieved (Figure 6), all from the same source [29], and a similar computational routine was carried out (see computational details section for a full description).

**Figure 7:**
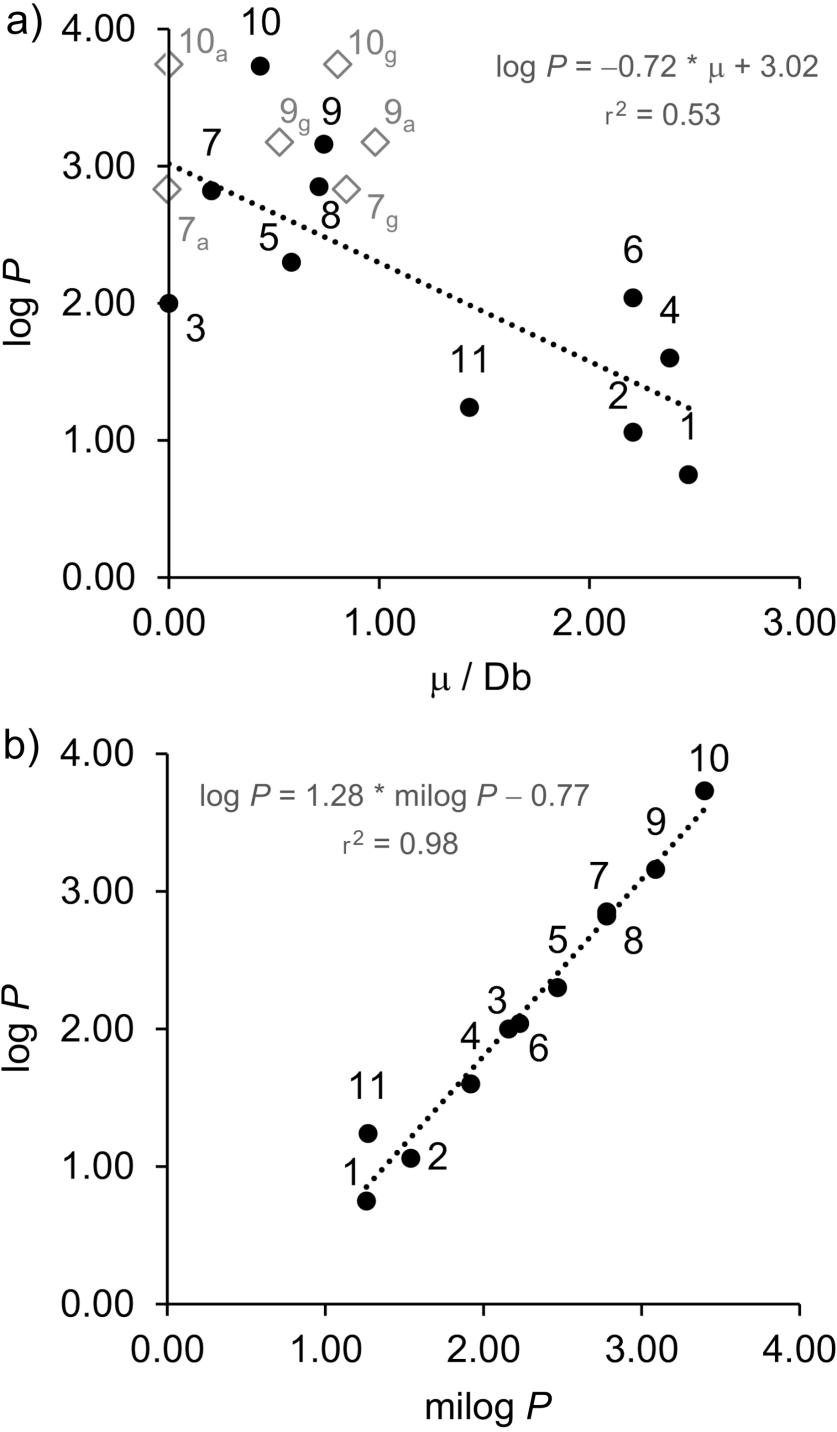
Correlation between the experimental log *P* of compounds **1**–**11** and a) dipole moment (for **7**, **9**, and **10**, μ is used as a weighted average of the *gauche* and *anti*-conformers), and b) predicted log *P* (milog *P*).

Compounds **7**, **9**, and **10** have the dependence of the molecular dipole moment μ with the rotation around the C–C bond. The correlation plots between the experimental log *P* of compounds **1**–**11** and a) dipole moment and b) predicted log *P* are shown in Figure 7.

From Figure 7a one can observe an r^2^ value of 0.53, which indicates a rough correlation between μ and log *P*. However, when applying the same hypothesis test, as for the first data set, a *p*-value of 0.0115 was obtained, which suggests that, although smaller than the first correlation, there is also a dependence between the calculated dipole moment and lipophilicity for small molecules. The considerably lower correlation may be explained by the fact that, in a small molecule, subtle structural changes strongly influence the overall molecular dipole and, sometimes, the introduction of a polar bond in a molecule decreases the overall molecular dipole moment. For instance, carbon tetrafluoride has four polar bonds, but it is apolar, while the other fluorinated methanes are all polar. Thus, the use of calculated molecular dipole moments as descriptors for lipophilicity in these small organofluorines should be used with caution. On the other hand, Figure 7b reveals a satisfactory correlation between the predicted and experimental values of log *P*, which reinforces that for simple molecules there are well-parameterized models for log *P* prediction, in spite of the remarkable systematic error.

## Conclusion

In summary, the overall molecular polarity is influenced not only by the nature of the substituent group (as considered by additive techniques of prediction), but also by the orientation of the neat molecular dipole moment vector. In this sense, methods that ignore such influences result in reduced accuracy when predicting physicochemical properties of complex structures, such as the herbicides presented herein. Taking into account the correlation between lipophilicity and molecular conformation contributes to rationalize the effect of fluorine introduction on lipophilicity. Furthermore, the use of the molecular dipole moment that considers information on the molecular conformation, as presented herein, is a simple and straightforward parameter that can be valuable as a descriptor in quantitative structure–property relationships (QSPR). This could contribute significantly in studies involving organofluorine agrochemicals, for example, towards modeling herbicidal activity, and/or for environmental risk assessment.

## Computational Details

The conformational search of agrochemicals **I**–**VII** was performed at the ωB97X-D/6-31G(d,p) [16–17] level of theory using the Spartan’18 software [30]. The lowest energy minimum conformation of each compound was then reoptimized and the dipole moment determined using a higher level of theory, ωB97X-D/6-311++G(d,p) [16,18], in the Gaussian 09 software [31]. The geometries and dipole moments for compounds **1**–**11** were calculated at the same level, which has been successfully applied to predict the conformational energies of other fluorine-containing compounds [32–34]. Frequency calculations were performed to confirm that the optimized geometries were true energy minima (no imaginary frequency) and to estimate thermodynamic energies, at 298.15 K. Solvent effects were accounted for by geometry optimization using the integral equation formalism variant of the polarizable continuum model (IEFPCM) [35]. Insights into the intramolecular interactions governing conformational preferences were obtained through the natural bond orbital (NBO) analysis [19,36]. The predicted log *P* was calculated using the Molinspiration Cheminformatics tool [25] and molecular structures were illustrated using CYLview [37].

## Supporting Information

File 1 Additional linear correlation, main conformers from the Monte Carlo conformational search, Cartesian coordinates and energies of the conformers of agrochemicals **I**–**VII** and compounds **1**–**11** analyzed herein.
