# Supplementary material for: Conformational preferences of fluorine-containing agrochemicals and their implications for lipophilicity prediction
Source: Beilstein J Org Chem. 2020 Oct 5;16:2469–76. doi: 10.3762/bjoc.16.200 (PMC7554678; doi:10.3762/bjoc.16.200)
Supplement: File 1 — Additional linear correlation, main conformers from the Monte Carlo conformational search, Cartesian coordinates and energies of the conformers of agrochemicals I–VII and compounds 1–11 analyzed herein. [file Beilstein_J_Org_Chem-16-2469-s001.pdf]

BEILSTEIN JOURNAL OF ORGANIC CHEMISTRY

Supporting Information

for

Conformational preferences of fluorine-containing agrochemicals and their implications for lipophilicity prediction

Daniela Rodrigues Silva, Joyce K. Daré and Matheus P. Freitas

Beilstein J. Org. Chem. **2020,** *16,* 2469–2476. doi:10.3762/bjoc.16.200

Additional linear correlation, main conformers from the Monte Carlo conformational search, Cartesian coordinates and energies of the conformers of agrochemicals I–VII and compounds 1–11 analyzed herein

License and Terms: This is a supporting information file under the terms of the Creative Commons Attribution License (https://creativecommons.org/licenses/by/4.0). Please note that the reuse, redistribution and reproduction in particular requires that the authors and source are credited.
The license is subject to the *Beilstein Journal of Organic Chemistry* terms and conditions: (https://www.beilstein-journals.org/bjoc)

Table of contents

Figure S1: Correlation between the experimental log *P* of agrochemicals **I–VII** and predicted log *P* (ACD/Labs).

Monte Carlo conformational search

Cartesian coordinates and energies of the conformers analyzed herein

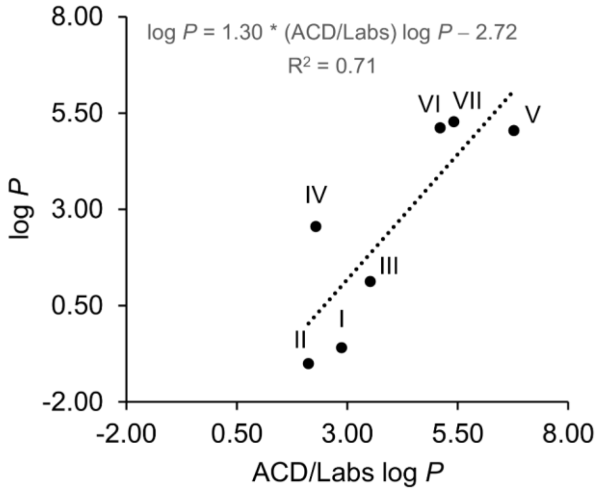

Figure S1: Correlation between the experimental log *P* of agrochemicals **I–VII** and predicted log *P* (ACD/Labs).

Monte Carlo conformational search

Table S1: Structures, relative conformational energies referenced to the global energy minimum (in kcal mol^{-1}) and Boltzmann population (in %) of the main conformations (i.e., those with a population ≥5%) of agrochemicals **I–VII** obtained in the gas phase conformational search at the ωB97X-D/6-31G(d,p) level of theory.

Comp.	Conformation	Δ*E*	Pop
**I**	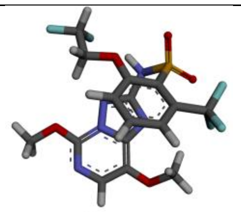	0.00	35
**I**	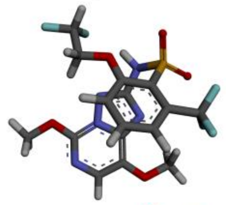	0.39	18
**I**	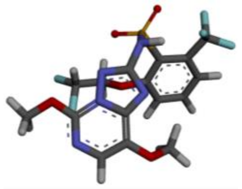	0.83	9
**I**	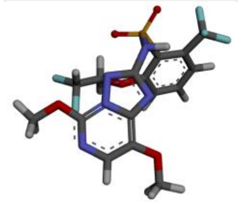	1.19	5
**II**	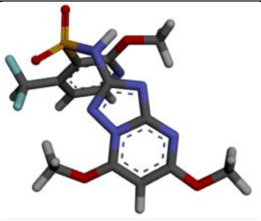	0.00	66
**II**	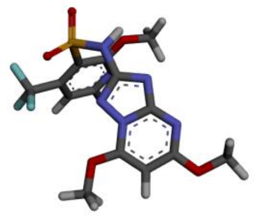	0.52	28

**II**	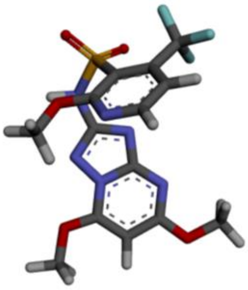	1.43	6
**III**	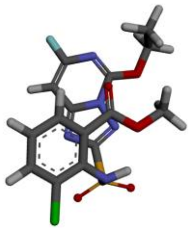	0.00	45
**III**	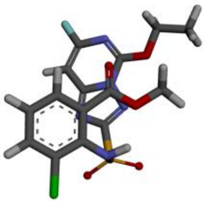	0.47	20
**III**	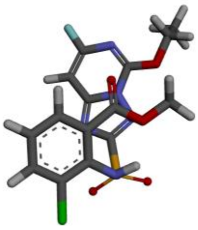	0.64	15
**III**	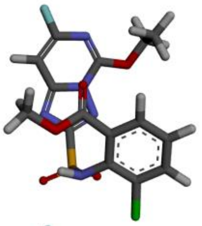	0.94	9
**III**	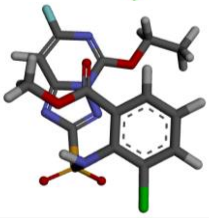	1.33	5
**IV**	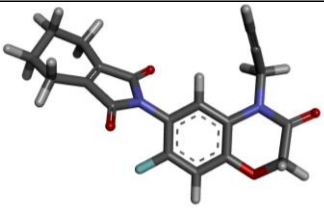	0.00	39
**IV**	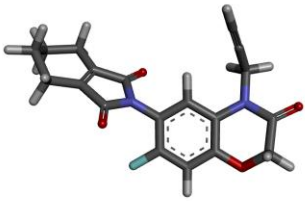	0.02	38

**IV**	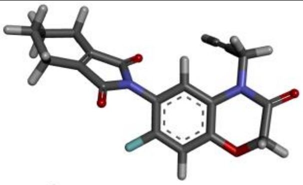	0.74	11
**IV**	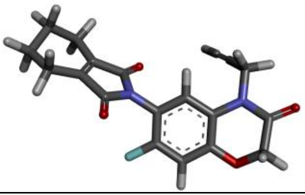	0.74	11
**V**	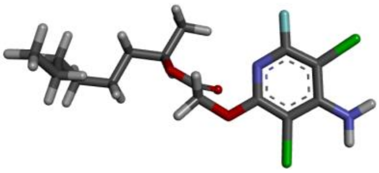	0.00	43
**V**	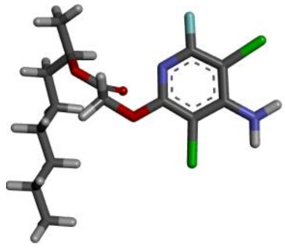	0.92	9
**V**	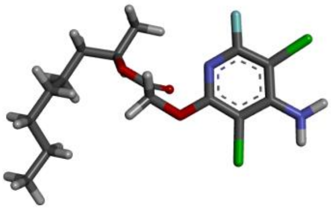	1.01	8
**V**	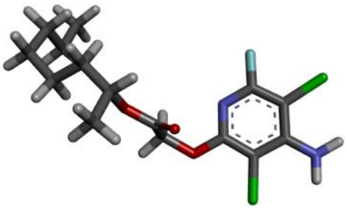	1.05	7
**V**	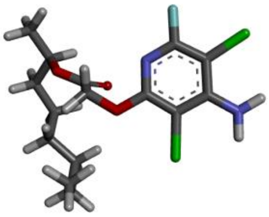	1.15	6
**VI**	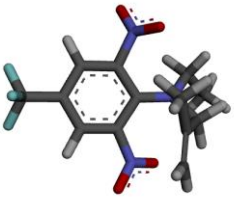	0.00	35

**VI**	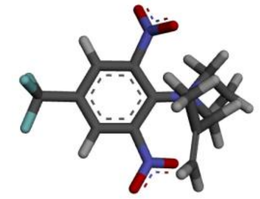	0.07	31
**VI**	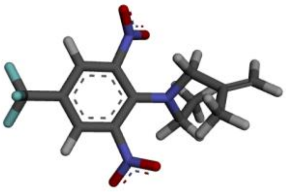	0.36	19
**VI**	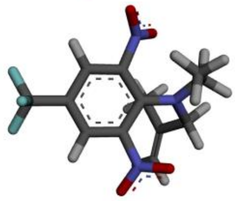	1.12	5
**VII**	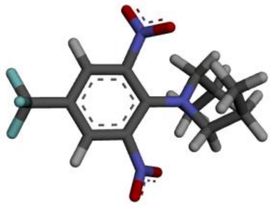	0.00	18
**VII**	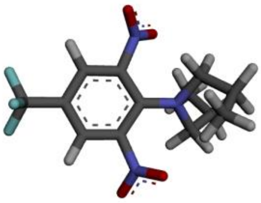	0.03	17
**VII**	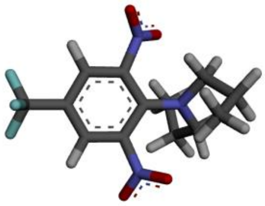	0.36	10
**VII**	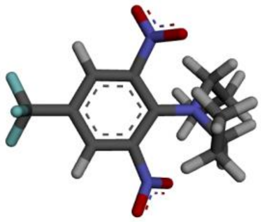	0.38	9
**VII**	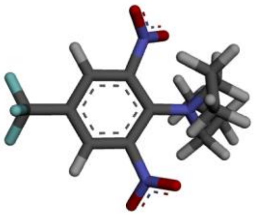	0.41	9

**VII**	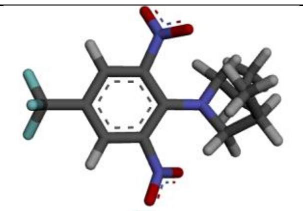	0.52	7
**VII**	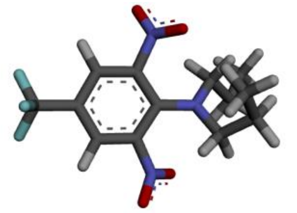	0.53	7

Cartesian coordinates and energies of the conformers analyzed herein

Cartesian coordinates (Å), energies (hartree), and the number of imaginary frequencies of the conformers of agrochemicals **I**–**VII** and compounds **1**–**11** analyzed herein at the ωB97X-D/6-311++G(d,p) level of theory.

Agrochem. $\mathbf{I_{gg}}$:

C	-1.24517900	-1.80516000	-0.83753500
C	-1.51138600	-3.16716300	-0.52113500
C	-2.73107400	-3.42607700	0.04038300
C	-3.41500900	-1.24521400	0.02691800
C	-0.60733100	0.10720100	-1.37398100
H	-2.99047400	-4.44746400	0.29202100
O	-4.23329000	-0.23357800	0.26489100
O	-0.66546400	-4.17563700	-0.79861300
N	-3.67444000	-2.47691700	0.30453600
N	-2.22382400	-0.88635300	-0.53665000
N	-1.80595900	0.36200200	-0.86641700
N	-0.21436300	-1.18116800	-1.38491600
C	-5.50176400	-0.57093700	0.83232500
H	-6.02370700	0.37686600	0.94018900
H	-6.05120400	-1.23977000	0.16851600
H	-5.37037000	-1.05374200	1.80191700
C	0.73206900	-3.93782300	-0.59365000
H	1.21348000	-4.90491400	-0.72285600
H	1.12139900	-3.21756400	-1.31184500
H	0.90822400	-3.57157700	0.42301700
N	0.20975800	1.14340000	-1.80466300
H	-0.22559900	2.05329400	-1.68516100
S	1.80847600	1.19479100	-1.24250500
O	2.54033900	0.16452000	-1.92029500
O	2.19200200	2.57972800	-1.32567800

C	1.59882600	0.68762600	0.49395100
C	0.56460900	1.31778000	1.20876100
C	2.29082000	-0.37487300	1.09295100
C	0.13853500	0.80702600	2.43202700
C	1.87036300	-0.85621100	2.32826600
C	0.78256800	-0.28928700	2.97342500
H	-0.68528200	1.26653800	2.96133700
H	2.40649200	-1.67052700	2.79407100
H	0.45320000	-0.69004600	3.92463400
C	3.54496400	-1.02106300	0.51191000
F	4.38846200	-0.11858600	0.01282800
F	3.28046800	-1.93972600	-0.41838000
F	4.22152800	-1.66685300	1.48815800
O	0.02200900	2.41634900	0.64865600
C	-1.24349400	2.86527300	1.07586200
H	-1.98426800	2.06489400	0.97627400
H	-1.21286600	3.24230300	2.10401100
C	-1.64483800	4.01246600	0.17571300
H	-2.67347500	4.32893000	0.36838400
F	-1.55670100	3.64573400	-1.13443600
F	-0.82711200	5.07798000	0.35333100

Total energy = -2165.255461
Number of imaginary frequencies = 0

Agrochem. $\mathbf{I}_{\mathbf{ag}}$:

C	0.82313800	1.97944300	-0.81608400
C	0.83705500	3.36206100	-0.47878000
C	2.00503200	3.84067400	0.04798100
C	3.09079700	1.83118200	-0.03208200
C	0.54792700	-0.01078500	-1.37426700
H	2.07576400	4.88930700	0.31107700
O	4.09222500	0.99005800	0.15260500
O	-0.19337000	4.19722900	-0.70608500
N	3.12120300	3.08646400	0.26103100
N	1.96768300	1.26027600	-0.56061800
N	1.78604300	-0.03980400	-0.90322800
N	-0.08635600	1.17697300	-1.34812800
C	5.30511800	1.55370000	0.65853100
H	5.99983100	0.71978500	0.72218200
H	5.68147900	2.31708800	-0.02392400
H	5.13977800	1.99843500	1.64098900
C	-1.51344200	3.69307600	-0.47869500
H	-2.17142400	4.55706000	-0.54709700
H	-1.78672700	2.94555600	-1.22203200
H	-1.58606000	3.25461500	0.52221800
N	-0.06107300	-1.18096300	-1.80889100
H	0.54820900	-1.98679200	-1.71140700
S	-1.61285100	-1.55147500	-1.23802000
O	-2.54446200	-0.70518500	-1.92421600
O	-1.70280200	-2.98835400	-1.30522300
C	-1.50830100	-0.98952700	0.48906500
C	-0.37229900	-1.39951300	1.20854900
C	-2.38427800	-0.06229000	1.06995600

C	-0.03698800	-0.78742100	2.41274100
C	-2.05321200	0.51740700	2.28981000
C	-0.87263400	0.18273600	2.93462800
H	0.86435400	-1.07156600	2.93955600
H	-2.73149600	1.22585500	2.74331000
H	-0.61830400	0.66232100	3.87225200
C	-3.74204600	0.32345300	0.49023000
F	-4.40379100	-0.73347600	0.02002300
F	-3.66442700	1.25401400	-0.46138400
F	-4.52026500	0.85053700	1.46203400
O	0.35000700	-2.39865900	0.66585000
C	1.67599600	-2.63391400	1.08807700
H	2.29395200	-1.74204700	0.94264800
H	1.71476500	-2.97607100	2.12740800
C	2.18478300	-3.74887700	0.19918800
H	1.57669700	-4.65471500	0.24364500
F	3.45602000	-4.04779900	0.57201400
F	2.21909000	-3.33494400	-1.09826700

Total energy = -2165.257772
Number of imaginary frequencies = 0

Agrochem. $\mathbf{I_{ga}}$:

C	-1.14331200	-1.86663600	-0.62430500
C	-1.30695900	-3.18075800	-0.10623100
C	-2.56637400	-3.50436800	0.32115300
C	-3.47120600	-1.46679400	-0.18111600
C	-0.63617500	0.00583100	-1.38774500
H	-2.74990500	-4.49478600	0.72022200
O	-4.41658200	-0.54807000	-0.25323800
O	-0.32662500	-4.10124200	-0.07064400
N	-3.63666900	-2.66192300	0.27463900
N	-2.24854500	-1.04821400	-0.62342800
N	-1.91884500	0.17828200	-1.10560200
N	-0.10971800	-1.20392600	-1.12358100
C	-5.72070300	-0.94368200	0.18242300
H	-6.34479800	-0.06594100	0.03459800
H	-6.08263800	-1.78152200	-0.41501200
H	-5.69975000	-1.23153400	1.23453700
C	1.00760800	-3.65206500	0.18895500
H	1.58498300	-4.55618800	0.37151100
H	1.41481000	-3.10515600	-0.66023300
H	1.03107800	-3.01370200	1.07855000
N	0.11310900	1.05746800	-1.88673800
H	-0.36680400	1.94740000	-1.83604800
S	1.72676400	1.25182100	-1.42580300
O	2.49045400	0.26760300	-2.13388800
O	1.97355500	2.66380100	-1.57445300
C	1.73723800	0.82413400	0.34281200
C	0.71620000	1.35879800	1.15153900
C	2.69866400	-0.01637600	0.92665500
C	0.62595900	1.00653900	2.49667300
C	2.58689400	-0.35873800	2.27022900
C	1.55164500	0.13888400	3.04239900

H	-0.15416800	1.41999300	3.12089400
H	3.32931200	-1.00277600	2.71789900
H	1.47947300	-0.13289400	4.08865900
C	3.92516200	-0.57450000	0.20701700
F	4.57794800	0.36395800	-0.47764300
F	3.63132000	-1.59322200	-0.60222100
F	4.80880000	-1.06039600	1.10613800
O	-0.16072800	2.20497300	0.57096700
C	-1.33800600	2.55977700	1.26545500
H	-1.81668800	1.67780300	1.70272200
H	-1.12775600	3.30004600	2.04462900
C	-2.30576800	3.15144400	0.26118200
H	-2.59910800	2.44469100	-0.51658400
F	-1.77438800	4.25721200	-0.31854800
F	-3.41783400	3.53557800	0.95082500

Total energy = -2165.256929
Number of imaginary frequencies = 0

Agrochem. **II**:

C	-1.99701300	0.30844900	2.48681000
C	-2.33559000	0.31751200	1.13492600
C	-1.91355500	-0.74228300	0.33340400
C	-1.22192800	-1.79975800	0.97160800
C	-1.25215800	-0.74358600	2.98181700
H	-2.31113400	1.10857300	3.14009500
H	-0.94985700	-0.76145000	4.02414600
N	-0.87852700	-1.78519500	2.24661100
O	-0.89252000	-2.85644000	0.23385900
C	-0.00957100	-3.82802700	0.80133000
H	-0.44342000	-4.26468000	1.70070100
H	0.11144800	-4.58175200	0.02675200
H	0.95179200	-3.36712600	1.03250700
S	-2.11363800	-0.83057400	-1.46649500
O	-2.95919800	-1.94735500	-1.80318600
O	-2.37626900	0.49418200	-1.95253800
N	-0.57955900	-1.26970300	-1.99781600
H	-0.48741700	-2.27780200	-2.00974800
C	0.55027900	-0.66296900	-1.46069500
C	2.48879300	-0.49148000	-0.69930900
C	2.42224600	1.83487200	-0.08677000
C	3.69286800	1.67388700	0.39424200
C	4.29619000	0.39695700	0.29079800
H	4.21830600	2.50880500	0.83107900
N	0.57093500	0.61246500	-1.12764600
N	1.66415800	-1.38639800	-1.22752200
N	1.83553400	0.72916300	-0.63467100
N	3.73167700	-0.66234500	-0.24233600
O	1.78031300	2.98154900	0.02413700
O	5.53224400	0.30892200	0.77439900
C	0.89189900	3.44400200	-1.01795300
H	-0.05842100	2.91962800	-0.97698300
H	0.76857600	4.50569000	-0.81769500
H	1.35685600	3.29885000	-1.99473400

C	6.19613300	-0.95058400	0.66616600
H	7.17205500	-0.79702100	1.12143500
H	5.64174400	-1.72599200	1.19709500
H	6.30129000	-1.24034300	-0.38061100
C	-3.17867300	1.49806600	0.65698600
F	-2.45911000	2.39368100	-0.02136700
F	-4.21550100	1.10940700	-0.08142800
F	-3.69419000	2.15476500	1.71613000

Total energy = -1943.511695
Number of imaginary frequencies = 0

Agrochem. **III**:

S	-1.88784000	-1.28379500	-1.50693300
N	-2.34867200	-1.22622300	0.11041500
H	-1.85524900	-1.96505200	0.60047200
C	-2.30095500	0.01249800	0.80581400
C	-3.38561900	0.89225000	0.73476900
C	-1.17463200	0.40159500	1.55782400
C	-3.33210900	2.14076500	1.34254600
C	-1.13140800	1.65507100	2.15982800
C	-2.20269400	2.52561400	2.04617300
H	-4.18784500	2.79937400	1.26370800
H	-0.24339100	1.93205300	2.71421000
H	-2.16599800	3.50054200	2.51668400
O	-1.65648500	-2.67967100	-1.77545300
O	-2.77398000	-0.47042900	-2.28875400
Cl	-4.85249200	0.43223500	-0.06486100
C	0.03523300	-0.45481900	1.76386900
O	1.14650800	-0.01050300	1.91620200
O	-0.24427900	-1.76069500	1.80726100
C	0.87725000	-2.64973200	1.91232200
H	1.42232600	-2.45377000	2.83650100
H	0.45017100	-3.64947700	1.92361700
H	1.52878200	-2.51788900	1.04815300
C	-0.30267300	-0.43178400	-1.48260400
C	1.06882400	1.12836300	-1.42924100
C	2.97028600	-0.10507500	-0.63088200
C	1.84890500	2.30173300	-1.40990800
C	3.12875000	2.12484300	-0.98426600
H	1.44201000	3.26040200	-1.69006600
N	0.77946300	-1.07299200	-1.09143300
N	-0.20471100	0.89151700	-1.70512600
N	1.67523500	-0.05820200	-1.06547300
N	3.69241900	0.97125100	-0.59890700
F	3.94542100	3.16873800	-0.91619400
O	3.38778200	-1.29101800	-0.27971800
C	4.69448600	-1.39270300	0.34055700
H	5.39476300	-0.77211300	-0.21937200
H	4.95082500	-2.44250400	0.20771100
C	4.63111500	-1.00351000	1.80132100
H	5.61067000	-1.16689400	2.25733200
H	4.36733900	0.04812300	1.91760200
H	3.89356600	-1.60829800	2.33140200

Total energy = -2187.404926
Number of imaginary frequencies = 0

Agrochem. **IV**:

C	0.40797200	-2.08873100	-0.22931200
C	1.73450200	-2.47270100	-0.26280800
C	2.71274700	-1.49400600	-0.23171500
C	2.37302600	-0.13758200	-0.16955500
C	1.03431900	0.21618800	-0.08943200
C	0.04118200	-0.75542000	-0.12647200
H	2.00830500	-3.51721200	-0.32800600
H	0.73557100	1.25216100	-0.00153700
C	4.90012500	-1.00193000	0.38131700
H	5.91192300	-1.28495200	0.10272900
N	3.41086800	0.81774500	-0.23755400
C	3.11655600	2.21601700	-0.52632700
H	4.04948700	2.67549300	-0.85525400
H	2.40119600	2.26012100	-1.35198600
C	2.59642400	2.94712300	0.63143400
C	2.16233700	3.54069300	1.57799400
H	1.78685700	4.06775000	2.42246000
C	4.70700100	0.46530800	0.04315200
O	5.62856800	1.25023300	0.05397100
O	4.01592600	-1.86359900	-0.31491300
H	4.77533500	-1.12796100	1.46527100
C	-3.40694900	0.45580600	-0.61470500
C	-3.52704500	-0.13494600	0.57494400
C	-4.50620400	1.15486100	-1.33862200
C	-4.78301700	-0.20739000	1.37308500
C	-5.67066000	1.41270900	-0.37221300
H	-4.13302500	2.08546800	-1.77499300
C	-5.97740200	0.18159800	0.49035700
H	-4.90373400	-1.21026000	1.79181800
H	-6.55946500	1.71008600	-0.93379400
H	-6.22795800	-0.66076700	-0.16467300
C	-1.99417200	0.32237100	-1.08298400
C	-2.19972900	-0.69378600	0.97674400
O	-1.50165600	0.73934600	-2.09652800
O	-1.90806700	-1.27576800	1.98455000
N	-1.32122500	-0.38725600	-0.07655000
F	-0.54232700	-3.02750700	-0.29621000
H	-4.83124200	0.53404600	-2.18205200
H	-5.41196300	2.25337500	0.28204600
H	-6.85405600	0.36837700	1.11502500
H	-4.69803800	0.46962900	2.23164000

Total energy = -1243.183078
Number of imaginary frequencies = 0

Agrochem. **V**:

C	-1.96248800	0.85130600	0.38900900
C	-3.25337300	1.28372900	0.10432200

C	-4.30185800	0.35373200	0.08854600
C	-3.96611100	-0.98685400	0.37030400
C	-2.64831600	-1.28056900	0.64469700
N	-5.57937600	0.72183900	-0.16435600
H	-5.76348900	1.65123500	-0.50045600
H	-6.27714000	0.01318000	-0.30891800
N	-1.67028800	-0.41061000	0.66443500
Cl	-5.18779300	-2.22021000	0.36075700
Cl	-3.57257500	2.95379800	-0.24669600
F	-2.31081600	-2.53721300	0.91454300
O	-0.98285100	1.76295500	0.39756500
C	0.33560600	1.28222600	0.54157200
H	0.96176700	2.17005000	0.63648100
H	0.44599600	0.66554500	1.43469000
C	0.78470700	0.49035700	-0.67710300
O	1.93588400	-0.11969500	-0.40552300
O	0.19907800	0.45041000	-1.72322400
C	2.52788100	-0.94488400	-1.44451200
H	2.32507800	-0.45834700	-2.40243600
C	4.02031900	-0.96574700	-1.16390700
H	4.17360300	-1.38139400	-0.16171800
H	4.48488400	-1.66554800	-1.86847700
C	4.68073500	0.40500000	-1.28569000
H	4.51078300	0.79505200	-2.29595800
H	4.18453300	1.10179700	-0.60319500
C	6.18653300	0.39243400	-1.00783700
H	6.68976200	-0.20740400	-1.77567000
H	6.56824200	1.41397300	-1.12010200
C	6.58682800	-0.13706700	0.37326700
H	6.37523100	-1.21137400	0.44000400
H	7.67373500	-0.04170900	0.48069100
C	5.90848000	0.57728200	1.54164400
H	6.07132400	1.65797100	1.44666600
H	4.82471600	0.42483000	1.48857100
C	6.41735800	0.09548400	2.89720800
H	5.91455300	0.60884000	3.72087800
H	6.24557100	-0.97847800	3.02008400
H	7.49252300	0.27170200	3.00009200
C	1.88105800	-2.31753900	-1.41055900
H	2.05037500	-2.79637500	-0.44265600
H	0.80654200	-2.24180700	-1.58368200
H	2.31159900	-2.94912800	-2.19168800

Total energy = -1939.678545
Number of imaginary frequencies = 0

Agrochem. **VI**:

C	-1.64910500	1.18403600	0.00066100
C	-0.28006900	1.32498500	-0.03516200
C	0.62311500	0.25562700	-0.26625800
C	-0.01765600	-0.99345900	-0.43968200
C	-1.38876600	-1.16826300	-0.32360000
C	-2.21324400	-0.07996000	-0.11751300
H	-2.26409900	2.06028800	0.16697800

H	-1.79642700	-2.16236600	-0.44882600
N	0.70376400	-2.17909100	-0.92788200
O	0.28895600	-3.26724600	-0.58575900
O	1.62820800	-2.00305500	-1.69802100
N	1.97356500	0.44688800	-0.28349600
C	2.91594600	-0.47588000	0.34977200
H	3.27746700	-1.23047000	-0.35415800
H	3.77398600	0.14640000	0.63273600
C	1.90475700	-0.21401600	2.69470100
H	1.01657400	0.34529600	2.38940100
H	1.66315600	-0.77586500	3.59807200
H	2.66672400	0.53268600	2.93885400
C	2.56997300	1.56282800	-1.01679700
H	3.10327100	2.21757600	-0.31907700
H	1.77032100	2.15072400	-1.46960500
C	3.49626200	1.08328300	-2.13066200
H	4.36460800	0.55147900	-1.73626800
H	2.96508800	0.41506800	-2.81108000
C	-3.70238700	-0.23579500	0.00123700
F	-4.35184900	0.61925200	-0.80603500
F	-4.12492900	0.01933100	1.25302100
F	-4.10602900	-1.47309600	-0.31050300
N	0.20864100	2.67206200	0.31761200
O	-0.31886300	3.61341600	-0.23991500
O	1.06462700	2.75634300	1.17267800
H	3.86260600	1.94499800	-2.69364300
C	2.38903400	-1.12912400	1.60502200
C	2.42785500	-2.45211900	1.73718900
H	2.10565100	-2.93648800	2.65263100
H	2.77684100	-3.09624700	0.93668000

Total energy = -1268.235534
Number of imaginary frequencies = 0

Agrochem. **VII**:

C	1.72578800	-1.18006000	-0.16005700
C	0.34575200	-1.17613900	-0.10002100
C	-0.44198700	0.00038600	-0.00718300
C	0.34341800	1.17994100	0.06957500
C	1.72403800	1.18922900	0.10186700
C	2.42973100	0.00601500	-0.03853000
H	2.24047700	-2.12754300	-0.25936300
H	2.23740200	2.13917800	0.18210200
N	-0.25967000	2.52102800	-0.01938300
O	0.12174700	3.35588200	0.77597000
O	-1.05491400	2.72315700	-0.91380400
N	-1.80367000	-0.00095200	0.00428000
C	-2.56344400	0.97271600	0.79289600
H	-1.86076800	1.53139100	1.41366900
H	-3.05388900	1.69278900	0.13082500
C	-3.58022800	0.32759700	1.73477800
H	-4.12244300	1.15072000	2.21152300
H	-4.32722500	-0.23282200	1.16470800
C	-2.94339700	-0.56524400	2.79410800

H	-2.22805600	0.00057000	3.39945300
H	-3.70143300	-0.96978400	3.46851900
H	-2.40871300	-1.40211000	2.34167300
C	-2.57599500	-0.97702000	-0.76905400
H	-3.04842400	-1.70043600	-0.09756600
H	-1.88437100	-1.53122500	-1.40602400
C	-3.61541400	-0.33501700	-1.68804000
H	-4.16573300	-1.15970600	-2.15254700
H	-4.35105900	0.22362100	-1.10178800
C	-3.00454500	0.55929200	-2.76129800
H	-2.30085000	-0.00483000	-3.38168000
H	-3.77817800	0.96132700	-3.41927200
H	-2.46262600	1.39777300	-2.32082200
C	3.93010400	0.00208200	0.00032900
F	4.44289700	1.14437400	-0.48056700
F	4.44168900	-1.00837000	-0.71760000
F	4.39437100	-0.13619400	1.25608300
N	-0.25060200	-2.51964000	-0.00565000
O	0.12183300	-3.35139000	-0.80845600
O	-1.03117300	-2.72676500	0.90058800

Total energy = -1269.472161
Number of imaginary frequencies = 0

Comp. **1**:

C	-0.06226700	0.00000000	0.35560500
C	1.37160000	0.00000000	-0.09041100
F	-0.71266300	1.09740300	-0.13533600
H	1.87578700	0.88888300	0.29241000
H	1.87578700	-0.88888300	0.29241000
H	1.41672800	0.00000000	-1.18103200
H	-0.19636500	0.00000000	1.44110600
F	-0.71266300	-1.09740300	-0.13533600

Total energy = -278.318378
Number of imaginary frequencies = 0

Comp. **2**:

C	-0.40834800	0.00393500	0.00000000
C	0.92205600	0.73661000	0.00000000
F	-0.55605100	-0.76852800	-1.07955600
H	0.98127800	1.35531700	-0.89749500
H	0.98127800	1.35531700	0.89749400
F	-0.55605100	-0.76852800	1.07955600
F	-1.40031900	0.91002600	0.00000000
F	1.95188700	-0.16784900	0.00000000

Total energy = -476.803130
Number of imaginary frequencies = 0

Comp. **3**:

C	0.77708800	-0.00001000	0.00000000

C	-0.77708800	0.00001000	0.00000000
F	1.22886400	-1.24895900	-0.00000200
F	1.22888600	0.62447300	-1.08151800
F	1.22888600	0.62446900	1.08152100
F	-1.22888600	-0.62447000	-1.08152000
F	-1.22888600	-0.62447100	1.08151900
F	-1.22886400	1.24895900	0.00000100

Total energy = -675.296225
Number of imaginary frequencies = 0

Comp. **4**:

C	-0.34421300	-0.06048500	0.00000000
C	-1.07312800	1.25416000	0.00000000
F	-0.67344000	-0.79493900	1.08136200
F	-0.67344000	-0.79493900	-1.08136200
H	-0.80728100	1.82234200	-0.89044500
H	-0.80728100	1.82234200	0.89044500
H	-2.14566400	1.05230200	0.00000000
Cl	1.43448200	0.14411100	0.00000000

Total energy = -737.926067
Number of imaginary frequencies = 0

Comp. **5**:

C	-0.45338100	0.45829600	0.00000000
C	0.94633500	-0.22390600	0.00000000
F	-0.54693400	1.23314600	1.08198200
F	-0.54693400	1.23314600	-1.08198200
Cl	-1.75388200	-0.73107400	0.00000000
F	1.88757200	0.71519500	0.00000000
F	1.09527400	-0.97841500	1.08192300
F	1.09527400	-0.97841500	-1.08192300

Total energy = -1035.646536
Number of imaginary frequencies = 0

Comp. **6**:

C	0.00000000	0.32606700	0.08926300
C	0.00000000	1.50736400	-0.84806600
F	0.00000000	0.75612100	1.37244700
Cl	-1.46110000	-0.68195900	-0.13519000
H	-0.89190800	2.10680300	-0.66001400
H	0.00000100	1.16732400	-1.88272800
H	0.89190800	2.10680400	-0.66001300
Cl	1.46110000	-0.68195900	-0.13519000

Total energy = -1098.277782
Number of imaginary frequencies = 0

Comp. $\mathbf{7_a}$:

C	0.60401700	-0.49421800	0.00009300
C	-0.60401700	0.49421900	0.00009300
F	0.53661300	-1.26941500	-1.08183000
Cl	2.13497800	0.38718300	-0.00018400
Cl	-2.13497700	-0.38718300	-0.00018500
F	0.53668700	-1.26928800	1.08211700
F	-0.53661400	1.26941500	-1.08183200
F	-0.53668900	1.26928900	1.08211700

Total energy = -1395.996957
Number of imaginary frequencies = 0

Comp. **7**$_\mathbf{g}$:

C	-0.12062700	0.77196600	0.32737500
C	0.12062700	-0.77196600	0.32737500
F	1.05645100	1.39705900	0.35328300
Cl	-1.05645100	1.30308500	-1.06907400
Cl	1.05645100	-1.30308500	-1.06907400
F	-0.77863800	1.07927300	1.44783000
F	-1.05645100	-1.39705900	0.35328300
F	0.77863800	-1.07927300	1.44783000

Total energy = -1395.996642
Number of imaginary frequencies = 0

Comp. **8**:

C	0.41828500	0.00000000	0.30745800
C	-1.08525300	0.00000000	-0.10702300
Cl	1.21101300	1.45870600	-0.29764100
F	0.46198800	0.00000000	1.64860400
F	-1.21624300	-0.00000100	-1.42758400
F	-1.68801800	1.07757000	0.38488900
F	-1.68801800	-1.07756900	0.38489100
Cl	1.21101300	-1.45870600	-0.29764100

Total energy = -1395.999748
Number of imaginary frequencies = 0

Comp. **9**$_\mathbf{a}$:

C	0.68276600	0.00004000	0.27518000
C	-0.87818400	-0.00014800	0.41809800
Cl	1.23677400	1.45955500	-0.55269100
F	1.17532800	-0.00002400	1.52426300
F	-1.23469500	1.07736100	1.11998000
F	-1.23450100	-1.07787000	1.11979100
Cl	1.23707100	-1.45931100	-0.55300100
Cl	-1.71988500	0.00007600	-1.13171800

Total energy = -1756.349318
Number of imaginary frequencies = 0

Comp. **9**$_g$:

C	0.58143400	0.14781200	0.30903900
C	-0.72840100	-0.53085400	-0.21650600
Cl	0.77953000	1.76002000	-0.38623100
F	0.48716100	0.24862600	1.64172200
F	-0.82296400	-1.74515800	0.32811000
F	-0.66561800	-0.66952600	-1.53984200
Cl	1.96961100	-0.87878100	-0.08900200
Cl	-2.16710700	0.40068900	0.21493200

Total energy = -1756.349596
Number of imaginary frequencies = 0

Comp. **10**$_a$:

C	-0.72179700	-0.00008000	0.31161300
C	0.72175200	-0.00004900	-0.31140700
Cl	-1.61055300	-1.44903900	-0.18925100
F	-0.60859200	0.00005500	1.64519100
F	0.60819300	-0.00019000	-1.64499400
Cl	-1.61089800	1.44885900	-0.18905300
Cl	1.61077100	1.44907400	0.18886100
Cl	1.61090800	-1.44877700	0.18926600

Total energy = -2116.701562
Number of imaginary frequencies = 0

Comp. **10**$_g$:

C	0.72021100	0.31626700	-0.25663600
C	-0.72021100	-0.31626700	-0.25663500
Cl	1.94620600	-0.96047400	-0.33875900
F	0.82211500	1.06325400	-1.36650500
F	-0.82211700	-1.06325400	-1.36650500
Cl	0.98296700	1.34909900	1.15277900
Cl	-0.98296700	-1.34909900	1.15278000
Cl	-1.94620500	0.96047500	-0.33875800

Total energy = -2116.701671
Number of imaginary frequencies = 0

Comp. **11**:

C	0.00000000	0.06553200	0.00000000
C	-0.00062500	1.38020000	0.00000000
H	-0.93837200	1.91398100	0.00000000
H	0.93685800	1.91451200	0.00000000
F	-1.07598400	-0.69510500	0.00000000
F	1.07657000	-0.69410500	0.00000000

Total energy = -277.068945
Number of imaginary frequencies = 0
